# An update on long-acting therapies in chronic sight-threatening eye diseases of the posterior segment: AMD, DMO, RVO, uveitis and glaucoma

**DOI:** 10.1038/s41433-021-01766-w

**Published:** 2022-01-01

**Authors:** Faruque Ghanchi, Rupert Bourne, Susan M. Downes, Richard Gale, Christina Rennie, Ian Tapply, Sobha Sivaprasad

**Affiliations:** 1 Bradford Teaching Hospitals NHS Foundation Trust, Bradford, UK; 2 Cambridge Eye Research Centre, Cambridge University Hospital, Cambridge, UK; 3 Oxford Eye Hospital, John Radcliffe Hospital, Oxford University NHS Foundation Trust, Oxford, UK; 4 York Teaching Hospital NHS Foundation Trust, University of York, York, UK; 5 Southampton Eye Unit, University Hospital Southampton NHS Foundation Trust, Southampton, UK; 6 Moorfields Eye Hospital, University College London, London, UK

**Keywords:** Therapeutics, Drug discovery

## Abstract

In the real-world setting, there is suboptimal compliance with treatments that require frequent administration and assessment visits. This undertreatment frequently has negative consequences in eye disease and carries a real risk to vision. For example, patients with glaucoma risk progression of visual loss even with a small number of missed doses, and patients with neovascular age-related degeneration (nAMD) who fail to attend a bi-monthly clinic appointment to receive an intravitreal anti-vascular endothelial growth factor (VEGF) drug injections may lose the initial vision gains in vision. Protracted regular treatment schedules represent a high burden not only for patients and families, but also healthcare professionals, systems, and ultimately society too. There has been a clear need for longer-acting therapies that reduce the frequency, and therefore the burden, of treatment interventions. Several longer-acting interventions for nAMD, diabetic macular oedema, retinal vein occlusion, uveitis and glaucoma have either been developed or are in late-phase development, some of which employ novel mechanisms of actions, and all of which of promise longer (≥3 month) treatment intervals. This review delivers an overview of anti-VEGF agents with longer durations of action, DARPins, bispecific anti-VEGF/Ang2 therapies, anti-PDGF and anti-integrin therapy, Rho-kinase inhibitors, the Port Delivery System, steroids, gene therapy for retina and uveitis, and for glaucoma, ROCK inhibitors, implants and plugs, and SLT laser and MIGS. The review also refers to the potential of artificial intelligence to tailor treatment efficacy with a resulting reduction in treatment burden.

## Abstract (zh)

在现实生活中, 患者的治疗依从性欠佳, 需要频繁进行治疗和评估回访。治疗不足通常会对眼部疾病产生负面影响, 并对视力造成威胁。例如, 青光眼患者即使遗漏少剂量药物使用, 也会有视力丧失的风险; 新生血管性年龄相关性退行性变 (nAMD) 的患者如果未能参加每两个月一次的复诊, 接受玻璃体内抗血管内皮生长因子 (VEGF) 药物注射, 可能会失去最初的视力增益。长期的定期治疗计划不仅对患者和家属, 而且对医疗专业人员、医疗系统乃至整个社会都是一个沉重的负担。

显然, 有必要采用长效疗法, 以减少干预的频率和负担。一些针对nAMD、糖尿病黄斑水肿、视网膜静脉阻塞、葡萄膜炎和青光眼的长效干预措施已经应用于临床或处于研究的后期阶段, 其中一些药物采用了新的作用机制, 所有这些干预措施都有望延长治疗间隔(≥3个月)。

本文对长效抗VEGF药物、DARPins、双特异性抗VEGF/Ang2治疗、抗PDGF和抗整合素治疗、Rho激酶抑制剂、Port传递系统、类固醇、视网膜和葡萄膜炎的基因治疗, 以及青光眼、ROCK抑制剂、眼植入物和栓子, 以及SLT激光和MIG进行了综述。该综述还提及了人工智能在调整治疗效果、减轻治疗负担方面的潜力。

## Introduction

The past two decades have seen significant advances in our ability to treat ocular disease, particularly those of the posterior segment. In particular, the advent of anti-vascular endothelial growth factor (VEGF) drugs has transformed the treatment of retinal diseases such as neovascular age-related macular degeneration (AMD), diabetic macular oedema (DMO), and macular oedema secondary to retinal vein occlusions (RVO). In glaucoma, the introduction of the prostaglandin analogue latanoprost, and more recently, the RhO-kinase (ROCK) inhibitor netarsudil and the nitric oxide (NO) latanoprostene bunod have resulted in significant therapeutic benefits for patients.

However, all of these therapies are associated with a relatively high treatment burden [1]: for example, intravitreally administered anti-VEGF therapy can involve hospital visits for assessment and anti-VEGF injections every 1–2 months. Given that many ocular diseases are age-related, and in 2019, the ‘baby boomer’ generation (those aged between 55 and 73 years) represented 21% of the population of the United Kingdom [2], the inevitable increase in age-related eye disease is associated with a heavy treatment burden for patients, their families, and the National Health Service.

Glaucoma is also largely a disease of an ageing population, and although the treatment is primarily topical therapy, this still requires strict compliance. It was estimated in 2020 that glaucoma represents the cause of moderate or severe visual impairment in 4.1 million people and blindness in 3.6 million [3]. Primary open-angle glaucoma (POAG) accounts for two-thirds of cases, with primary angle-closure glaucoma (PACG) the next most common form of the condition [4]. Most forms of glaucoma, including secondary glaucomas, are associated with a raised intraocular pressure (IOP) as the major causative risk factor. All current treatments target IOP to control disease progression, and treatment options include medical, laser or surgical therapies. The mainstay of glaucoma therapy remains medical treatment in the form of eye drops, although accurate continued compliance to prescribed treatment regimens has been found to be suboptimal, especially when compared with other chronic medical conditions [5].

The reality is that patients have busy lives, have other commitments, and other health concerns as they age. They may forget an appointment or to take their eye drops, and with anti-VEGF therapy, may miss injections because of reluctance to be a burden to the relatives or carers who bring them to the appointment, have needle-phobia, or simply become weary of repeated visits to an eye clinic. One approach that should help address these issues is the introduction of longer-acting therapies. Longer-acting therapies—particularly those that require fewer clinic visits for treatment administration—will result in a lower burden for patients (reducing the number of clinic visits means less time spent on travel and hospital appointments, with less disruption to work); for families and carers (who accompany the patient), as well as for overstretched healthcare systems. Therapies with extended durations of action will lessen the risk of forgotten or missed doses. In addition, novel treatments (e.g. gene therapies for inherited retinal disorders), will result in even greater demand for the services of eye care professionals, further increasing the pressure on already stretched health resources. The introduction of longer-acting therapies is therefore welcome and timely.

**Fig. 1:**
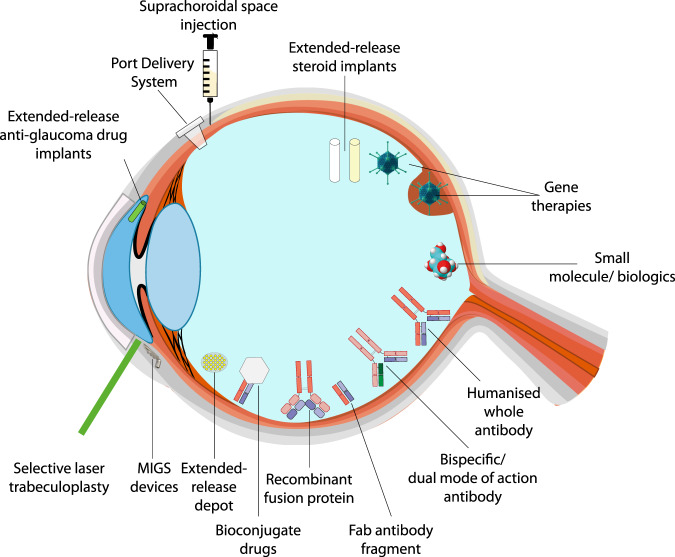
Schematic illustration of the types of ocular therapeutics and interventions that can deliver long therapeutic durations of action. For retinal disorders intravitreal injections are employed to deliver various antibodies, extended release depot preparations and implants, as well as new biologics and some gene therapy. Subretinal injections (after vitrectomy) are used to deliver gene therapy, suprachoroidal injection route is employed to deliver steroids and has potential for use with other agents. Port delivery system anchored at pars plana is used as a reservoir of therapeutic agent that dissolves in vitreous over time. For glaucoma the options include extended release implants in the anterior chamber, MIGS devices and selective laser trabeculoplasty.

For the purpose of this review article, we defined ‘long-acting’ therapies as those with a potential treatment effect of more than 12 weeks. We included glaucoma in this review, as longer durations of treatment effects would help resolve issues with patient compliance associated with topically administered anti-glaucoma drug regimens. Innovative drug delivery methods and routes are used to achieve the goal of longer duration of action in the eye (Fig. 1).

## AMD, DMO, RVO and uveitis

### Anti-VEGF therapy

Pegaptanib sodium (MacuGen, Pfizer, New York, NY, USA) was the first anti-VEGF agent approved for ophthalmic use but was superseded by other agents. The next anti-VEGF agent to be approved, ranibizumab, has a posology for neovascular age-related degeneration (nAMD), DMO, PDR and RVO at one injection per month until maximum visual acuity is achieved (or there are no signs of disease activity) and thereafter, the decision when to treat with the next dose is determined by the treating physician based on disease activity, as assessed visual acuity or anatomical parameters [6], a regimen called ‘treat-and-extend’ (Table 1). If disease activity recurs, then the dosing interval is shortened. There are no data on the use of intravitreal ranibizumab injections at 12 or more weekly intervals. Bevacizumab (Avastin, Roche, Basel, Switzerland), which is used to treat certain cancers, is frequently used off-label as an alternative to European Medicines Agency (EMA)-approved anti-VEGFs. Bevacizumab is supplied in a single-use vial (4 mL, 100 mg) for systemic use [7], and these doses are withdrawn from the vial under sterile conditions to give 1.25 mg doses in a volume of 0.05 mL. This approach, while time-consuming, can be very cost-efficient for a drug that is reasonably similar in its efficacy to the other anti-VEGFs currently available [8, 9]. There is no specific guidance for bevacizumab’s posology, but its initial studies in the eye employed a monthly dosing regimen, and later studies lent support for administration in treat-and-extend [10] and pro re nata (PRN) regimens [11]. However, treat-and-extend studies that compared bevacizumab to licensed anti-VEGF agents indicated a need for more frequent injections, as bevacizumab use was associated with less macular fluid resolution [12]. There are no studies comparing bevacizumab with current licensed anti-VEGF actions for a longer extension of 12 or more weeks. Aflibercept (Eylea, Bayer HealthCare, Berlin, Germany) is another anti-VEGF agent that also blocks the pro-angiogenic cytokine, placental growth factor (PIGF). Based on the evidence from Phase III trial programme [13–18], aflibercept’s approved posology involves administering the drug initially as three (nAMD) or five (DMO) monthly loading doses, followed by dosing every second month for the first year (or in patients with RVO, monthly treatment [typically ≥3 months] until maximal visual acuity gains are achieved). After this point, a treat-and-extend regimen is possible [19].

**Table 1:** Long-acting Anti-VEGF pharmacotherapies for nAMD, BRVO, CRVO and DMO.

**Anti-VEGF agent**	**Type**	**Mechanism**	**EMA-approved ophthalmic indication in adults**	**EMA-approved dosing regimen**
Aflibercept	Human recombinant fusion protein; combines the second Ig domain of VEGFR-1 and third Ig binding domain of VEGFR-2 with the constant Fc portion of IgG1	Soluble decoy receptor with high affinity for binding to VEGF-A and PlGF	nAMD, DMO, BRVO, CRVO, myopic CNV	nAMD: Initially q4w for 3 doses, then q8w, then T&E BRVO/CRVO: q1m DMO: Initially q4w for 5 doses, then q4w for rest of first year, then T&E
Brolucizumab	Humanised, single-chain variable fragment (scFv) antibody	Soluble decoy receptor with high affinity to neutralise all VEGF-A isoforms	nAMD	nAMD: Initially q4w for 3 doses, then q8w (if disease activity is present) or q12w (if disease activity is not present).
				**Investigated regimens**
Conbercept	Human recombinant fusion protein; second Ig domain of VEGFR-1 and the third and fourth Ig domain of VEGFR-2 with the Fc portion of human IgG1.	Soluble decoy receptor with high affinity for binding to VEGF-A and PlGF	N/A	Monthly for three doses, then q3m or T&E or PRN dosing for up to q12m
Abicipar	Designed ankyrin repeat protein (DARPin)-based therapy	Binds all VEGF isoforms	N/A	q12w dosing regimens were evaluated; development discontinued.
Faricimab	Bispecific antibody: modified Fc portion of humanised IgG with one anti-Ang2 Fab and one anti-VEGF-A Fab	Blocks VEGF-A and angiopoietin-2; modified Fc portion reduces both systemic absorption and potential for ocular inflammation	N/A	q4w, q8w, q12w and q16w dosing regimens and PDS under evaluation
KSI-301	Bioconjugate: humanised anti-VEGF monoclonal antibody and phosphorylcholine-based polymer added to prolong duration of molecule in eye	Blocks all VEGF isoforms	N/A	nAMD trials: q3m to q5m regimen evaluated.
RGX-314	Gene therapy, encodes an anti-VEGF Fab	AAV8 encoding anti-VEGF-A similar to Ranibizumab	N/A	Single dose (potentially)
ADVM-022	Gene therapy: encodes aflibercept	AAV encoding anti-VEGF-A similar to aflibercept	nAMD	Single dose (potentially)

*DMO* diabetic macular oedema, *BRVO* branch retina vein occlusion, *CNV* choroidal neovascularisation, *CRVO* central retinal vein occlusion, *Fab* fragment, antigen binding, *Ig* immunoglobulin, *m* month, *nAMD* neovascular age-related macular degeneration, *PDS* Port Delivery System, *PIGF* placenta growth factor, *PRN* pro re nata (as needed), *q* *quaque* (every), *R* receptor, *T&E* treat-and-extend, *VEGF* vascular endothelial growth factor, *w* weeks.

#### Anti-VEGF agents with proven longer duration of action

##### Aflibercept

Recently, two studies, ALTAIR [20] and ARIES [21], investigated aflibercept for the treatment of treatment-naïve patients with nAMD, and used a treat-and-extend regimen that extended treatment out to a maximum of 16-week (i.e. 4-month) intervals. Patients in both trials were divided into two groups: those who had their treatment interval extended by 2 weeks after every assessment, or those who had it extended by 4 weeks. In the ALTAIR study, the switch to treat-and-extend was after 3 monthly aflibercept injections in a mostly Japanese population, but in the ARIES (predominantly European descent participants) study, the switch to treat-and-extend was deployed either early (after the three initial monthly doses) or late (at the 52-week stage, before then, patients received aflibercept dosing every second month). In ALTAIR, the best-corrected visual acuity (BCVA) letter gains in the 2-week and 4-week extension groups were, respectively, +9.0 and +8.4 letters at 52 weeks, and +6.1 letters at week 96, with the last injection interval being ≥12 weeks in 42.3% and 49.6% of patients, respectively, at 52 weeks, and 56.9% and 60.2%, at 96 weeks, with the mean number of injections in both groups being 10.4 at this time point. The study authors noted that the ‘outcomes were similar between the 2- and 4-week groups’. The ARIES study investigators found that outcomes were fairly similar in the groups where patients switched to a treat-and-extend regimen early (week 104 BCVA change from baseline +4.3 letters; a mean of 12.0 injections; 47.2% of patients had last injection interval was ≥12 weeks) or late (+7.9 letter gain; a mean of 13.0 injections; 51.9% had a last injection interval of ≥12 weeks; mean of 13.0 injections).

Ziv-aflibercept (Zaltrap, Sanofi, Paris, France, indicated to treat colon cancer via systemic infusion) contains the same active molecule (aflibercept) but in a different buffer, and is supplied in 4 mL/100 mg and 8 mL/200 mg vials. Ziv-aflibercept can be prepared in a similar manner to bevacizumab for intravitreal injection, and its off-label use to treat nAMD, macular oedema secondary to RVO and DMO, is reviewed elsewhere [22]. Ziv-aflibercept has been investigated in two Phase I trials with PRN dosing (NCT04290195 and NCT02173873) and is being investigated with up to 12 weekly doing in one Phase 2 trial (NCT02173873), although none of these trials have reported results to date.

##### Brolucizumab

In 2020, brolucizumab (Beovu, Novartis Pharma AG), another humanised monoclonal single-chain antibody fragment that targets VEGF-A, was approved for the treatment of nAMD, and is currently undergoing late-phase clinical investigation for other retinal diseases, including PDR (NCT04278417), DMO (NCT04058067), and RVOs (NCT03802630 and NCT03810313). In nAMD, brolucizumab is administered monthly for the first three doses, thereafter the dosing regimen is decided by the treating physician based on visual acuity/anatomical parameters, with the EMA-approved posology being ‘in patients without disease activity, treatment every 12 weeks (3 months) should be considered. In patients with disease activity, treatment every 8 weeks (2 months) should be considered.’ The Phase III brolucizumab HAWK and HARRIER studies [23] that were instrumental in its approval for the treatment of nAMD showed that after three monthly loading doses, up to 50% of patients could be maintained on 12 weekly dosing intervals. It showed that as early as 16 weeks after commencement of therapy (after three injections), that it can be identified whether a patient requires 8-weekly or 12 weekly injections. However, ocular inflammation rates of 4.6% in the HAWK and HARRIER studies (intraocular inflammation: 3.3%, retinal vasculitis, 2.1%; 0.5% of these findings were associated with severe vision loss) [23] and post-marketing reports of intraocular inflammation, in particular, retinal vasculitis and vascular occlusion are of concern and have highlighted the importance of case selection as well as vigilance [24–29]. These concerns were highlighted by the release of the 1-year data from the Phase 3 MERLIN trial (NCT03710564) that compared 4-weekly regimens of brolucizumab and aflibercept in patients with nAMD and persistent retinal fluid despite anti-VEGF therapy. This trial met its primary efficacy endpoint of noninferiority to aflibercept in terms of VA gains and certain anatomical outcomes, but again showed that brolucizumab was associated with higher intraocular inflammation rates (9.3%) than aflibercept (4.5%) when administered using the same regimen (retinal vasculitis: 0.8% vs. 0.0%; retinal vein occlusion: 2.0% vs. 0.0%) [30]. The rates of vision loss from any cause were 4.8% and 1.7%, respectively. Following this, Novartis announced the termination of the MERLIN, branch RVO RAPTOR (NCT03802630) and central RVO RAVEN (NCT03810313) Phase 3 studies of brolucizumab and have advised Brolucizumab should not be used if less than q8w injections are needed after initial loading phase [30].

Nevertheless, multiple brolucizumab dosing regimens are currently under investigation for diabetic retinopathy in Phase 3 trials. These include three 6-weekly loading injections, followed by 12 weekly maintenance doses for PDR (NCT04278417); and for DMO, five loading doses with ‘subsequent doses per protocol-specified maintenance schedule’ up to 12 weekly dosing (NCT03481660; NCT04079231, NCT03481634) and monthly dosing (NCT03917472). Similarly, the TALON study (NCT04005352) is currently evaluating brolucizumab in extended treatment interval (treat-and-extend fashion) compared to aflibercept in patients with nAMD.

##### Conbercept

Conbercept (Lumitin, Chengdu Kanghong Biotech Co., Ltd., Sichuan, China), like aflibercept, is a VEGF receptor 1 and 2 fusion protein, which has undergone or is currently undergoing Phase III clinical evaluations for indications including nAMD, (NCT03577899, NCT03630952) DMO (NCT02194634), uveitic macular oedema (NCT04296838), and macular oedema following RVO (NCT03108352), but is currently only approved for the treatment of nAMD in China and Mongolia. The Phase III PANDA-1 trial that compared 8- and 12 weekly conbercept doses with 8-weekly aflibercept doses in patients with nAMD failed to meet its primary endpoint at 1 year (NCT03577899), and it and the PANDA-2 trial (NCT03630952) have now closed. The Phase 3 PHOENIX study, performed in patients with choroidal neovascularisation secondary to AMD, employed a dosing regimen of three monthly doses, followed by quarterly (q3m) dosing for the rest of the 12-month study period [31], and one Phase IV clinical trial (NCT02802657) is evaluating conbercept using both a treat-and-extend and a PRN regimen (where treatment intervals can be extended up to q12w).

Finally, another anti-VEGF agent, OPT-302 (Ophthea, South Yarra, Australia) which blocks the C and D isoforms of VEGF has completed a series of Phase I and II trials in wet AMD and DMO (NCT02543229, NCT03345082, NCT03397264). Phase III trials in nAMD (NCT04757610, NCT04757636) are currently underway, but only a 4-week dosing interval is currently being investigated.

##### Biosimilars

It is worth noting that there are multiple anti-VEGF ‘biosimilars’ in development (Table 2). Their duration of action compared to the parent compound is unclear. Currently, there are no data as to whether their action can be lengthened to 12 weeks or longer. Depending on the molecule, these trials are evaluating 4- or 8-weekly dosing regimens. However, even though these agents might be cheaper than existing treatment options, the concern is that they will not be able to help with existing capacity and compliance issues if their treatment intervals are not extendable.

**Table 2:** Anti-VEGF biosimilars in development^a^.

Company	Compound name	Registered clinical trials	Phase
Aflibercept biosimilars			
Amgen	ABP 938	NCT04270747	III
Alteogen	ALT-L9	NCT04058535	I
Coherus Biosciences	CHS-2020	–	
Formycon	FYB203	NCT04522167	III
Momenta/Mylan	MYL-1701P	NCT03610646, NCT04674800	III (both)
Samsung Bioepis	SB15	NCT04450329	III
Ranibizumab biosimilars			
Coherus Biosciences/Bioeq	FYB201	NCT02611778	III
Lupin	LUBT010	NCT04690556	III
Samsung Bioepis	SB11	NCT03150589	III
Xbrane	Xlucane	NCT03805100	III
Bevacizumab biosimilars			
Outlook therapeutics	ONS-5010	NCT03834753	III
Pfizer	Bevacizumab-bvzr/Zirabev	Currently only oncology indications	
Amgen/Allergan	Bevacizumab-awwb/MVASI	Currently only oncology indications	

^a^Duration of efficacy to be determined.

### DARPins

Abicipar pegol (Allergan/Abbvie, Dublin, Ireland), is a designed ankyrin repeat protein (DARPin)-based therapy that binds all VEGF isoforms [32]. It has a considerably longer half-life than ranibizumab in aqueous humour (13 vs. 7 days). A pooled analysis of two Phase III trials, CEDAR and SEQUOIA [33], has demonstrated its noninferiority to ranibizumab when administered at 12-week injection intervals compared with ranibizumab’s 4-week intervals [34]. Issues with ocular inflammation, thought to be related to the manufacturing process of the drug, were observed in CEDAR and SEQUOIA (the 1-year incidence of intraocular inflammation was 15.4% and 15.3% for abicipar dosed every 8 and 12 weeks, respectively). The manufacturing process was modified and abicipar was evaluated in patients (*n* = 123) with nAMD over a 28-week period in the open-label MAPLE study (NCT03539549). Patients received three monthly loading doses followed by 8-weekly doses, for a total of five injections. The incidence of intraocular inflammation was reduced to 8.9% [35]. To date, abicipar has not been approved by the US Food and Drugs Administration and its clinical development remains paused.

### Bispecific/dual action drugs

The bispecific antibody faricimab (Roche, Basel, Switzerland) targets not only VEGF-A, but also angiopoietin (Ang)-2. The Ang-tyrosine kinase endothelial receptor (Ang-Tie) pathway is responsible for regulating vascular homoeostasis through Tie-2 receptor, the breakdown of which leads to vascular permeability, inflammation, and angiogenesis. In a healthy state, Ang1 binds the Tie-2 receptor, the pathway becomes activated, and this maintains vascular health. Under the pathological conditions found in AMD or DMO, however, Ang2 acts as a competitive antagonist of Ang1, and inhibits the activation of the Tie-2 receptor, destabilising the retinal vasculature and making it more susceptible to the effects of pro-inflammatory cytokines and VEGF [36].

Faricimab has been evaluated in two Phase II trials (STAIRWAY [37] and AVENUE [38]) for the treatment of nAMD, and one Phase II study for the management of DMO (BOULEVARD [39]). AVENUE and STAIRWAY showed that faricimab, with either monthly dosing (AVENUE) or every 12 or 16 weeks (STAIRWAY) provided similar clinical outcomes to patients treated with 4-weekly ranibizumab [40]. The BOULEVARD study employed 4-weekly dosing, but the Phase III faricimab DMO trials (YOSEMITE, NCT03622580, and RHINE (NCT03622593) employed an 8–12 or 16 weekly dosing interval regimen (personalised to each patient regimen) compared with 8-weekly aflibercept dosing (after completion of the 3-month, monthly injection loading phase). One-year outcome results from RHINE and YOSEMITE (for DMO) and Tenaya and Lucerne (AMD) showed that faricimab met the primary outcome measures, and showed the majority (70%) of patients could be maintained at 12 weekly injections and nearly half could be maintained on 16 weekly dosing [41].

Another anti-VEGF/Ang2 bispecific antibody in development is BI 836880 (Boehringer Ingelheim Pharma GmbH & Co KG, Biberach, Germany), which is currently undergoing a Phase I dose-ranging clinical trial (NCT03861234) for the treatment of nAMD. The future might see more biphasic antibodies that target disease-causing or disease-exacerbating cytokines, with the development of a platform to create dual targeting fragment antigen-binding region (DutaFab) molecules. This approach has already been used to create DutaFabs that bind both VEGF-A and platelet-derived growth factor (PDGF)-BB with high affinity [42].

### Bioconjugate drugs

Another method of producing longer-acting therapies is to bind therapeutic molecules covalently to lipid or polymer carrier molecules in a process that results in ‘bioconjugate’ drugs [43]. One such bioconjugate, KSI-301 (KODIAK sciences, Palo Alto, CA, USA) is formed from a humanised anti-VEGF monoclonal antibody and a phosphorylcholine-based polymer, with the polymer acting to prolong the molecule’s duration in the eye following intravitreal injection [44]. A Phase I dose-ranging study (NCT03790852) included patients with nAMD, DMO or RVO and utilised a dosing regimen of three initial monthly doses, followed by retreatment as per the study protocol. Preliminary results show that 92% of eyes with nAMD eyes could be extended to ≥3 months after the last loading dose without receiving retreatment; in DMO, 72% of eyes could be extended to ≥4 months, and in RVO, half of all eyes could be extended to ≥3 months [45]. A Phase II trial, DAZZLE (NCT04049266) for patients with nAMD is currently ongoing where 12-, 16-, and 20-week treatment intervals are under investigation. Seventy-five percent or more patients could be maintained at 16 weekly intervals at 1 year, while 2 out of 3 patients across the three indications had treatment-free intervals of 6 months or more [46].

### Kinase/cytokine inhibitors

Tyrosine kinases are a family of enzymes that phosphorylate and activate numerous receptors. Tyrosine kinase inhibitor (TKI) drugs can therefore inactivate certain receptors (e.g. VEGF receptors) that are responsible for driving the pathologies involved in many retinal diseases. One such TKI is sunitinib maleate, which has activity against both VEGF-A and PDGF and is being developed as GB-102 (GrayBug Vision, Inc., Redwood City, CA, USA) for use as an nAMD and DMO therapy. The drug has an extended-release formulation; sunitinib is encapsulated within bioerodable and slowly degradable polymer nanoparticles designed to release clinically effective sunitinib concentrations over a 6-month period, meaning the drug can be administered by intravitreal injection every 6 months [47]. Results from the nAMD Phase I/IIa study, ADAGIO, showed that GB-102 remained effective in 88% and 68% of patients at 3 and 6 months after dosing, respectively [48]. ALTISSIMO, a Phase II trial in nAMD (NCT03953079), is currently ongoing and results are expected in 2021. Finally, a Phase II trial in patients with DMO or RVO (NCT04085341) completed in July 2020.

OTX-TKI (axitinib intravitreal implant, Ocular Therapeutix Inc., Bedford, MA, USA) is a dried polyethylene glycol-based hydrogel fibre-containing dispersed microcrystals of the small molecule tyrosine kinase inhibitor axitinib, which is designed to deliver therapeutic concentrations of the drug over the course of a year in order to enable 12-monthly dosing in patients with nAMD. Axitinib inhibits VEGF1-3, c-KIT and the PDGF receptor. Preliminary findings from a Phase I trial of OTX-TKI presented at the 2020 Retina Society meeting suggested that the durability of therapy ‘was up to 4.5 months’ and that the ‘implant biodegraded in all subjects in cohort 1 by 9–10.5 months’ [49]. The OASIS trial (NCT04626128) is a Phase I/II open-label, dose-escalation study looking at axitinib suspension injections in suprachoroidal space, and results are due to be available in late 2021. Finally, PanOptica Pharma (Mount Arlington, NJ, USA) is developing a topically applied TKI drop, PAN-90806, which reaches the retina via the trans-scleral vascular route to reach target tissues. It has been investigated in Phase I/II clinical trials for the treatment of nAMD (NCT03479372, NCT02022540) and proliferative diabetic retinopathy (NCT02475109). Nevertheless, its potential role, either as monotherapy or perhaps as an adjunctive therapy that might help reduce treatment intervals with other agents, still needs to be confirmed future studies.

### Anti-PDGF therapy

Several of the drugs mentioned above target PDGF, and this action is important, as the response to anti-VEGF therapy can wear off over time. This is because the new blood vessels start to become covered with pericytes that protect the new vessels and nourish them with several cell survival cytokines, including VEGF. PDGF is one molecule that recruits the pericytes to the vessels, and thus blocking PDGF could improve outcomes by enhancing the effect of anti-VEGF. Anti-PDGF therapy has been tried in combination with ranibizumab in the past as a naïve nAMD therapy. A 32-mer pegylated DNA aptamer that binds PDGF-BB and PDGF-AB homodimers and heterodimers agent pegpleranib (Fovista, Ophthotech, New York, NY, USA) was evaluated in a pair of Phase III trials (NCT01944839 and NCT01940900) but ultimately failed to show any superiority over ranibizumab alone [50]. Nevertheless, there is a solid mechanistic rationale for PDGF inhibition, and it could be that PDGF inhibition, in combination with the inhibition of VEGF and other cytokines, might be a strategy that may eventually prove its worth.

### Recombinant, human complement factor H therapy

GEM103 (Gemini Therapeutics, Cambridge, MA, USA) is a recombinant human complement factor H (CFH) molecule that is currently under Phase II clinical trial investigation for both neovascular (NCT04684394) and dry AMD (NCT04643886) using monthly and every other month dosing regimens. CFH plays a central role in regulating the immune system’s alternative pathway in terms of protecting host cells during an inflammatory/immune response. Over-activation of the innate immune system is thought to be one of the factors that can lead to the pathology in dry AMD. Lampalizumab (Roche) was a humanised Fab that binds to complement factor D, and 4-weekly and 6-weekly dosing regimens were under investigation in Phase III clinical trials for the treatment of geographic atrophy (GA; NCT02745119, NCT02247531, and NCT02247479). However, the trials were terminated due to failure of the drug to meet the trial’s primary endpoints.

Pegcetacoplan (Apellis Pharma, Waltham, MA, USA) a synthetic peptide-polyethylene glycol polymer conjugate that binds and inhibits complement proteins C3 and C3b. It is currently under Phase III clinical investigation for the treatment of GA secondary to AMD (NCT04770545, NCT03525600) using 4-weekly and 8-weeky dosing regimens. Twenty-four-month data from an earlier Phase 1b APL2-103 (NCT03777332) in 13 patients (where the eye with the worst BCVA received the study drug) revealed that GA lesions pegcetacoplan-treated eye had a growth rate that was 46% lower than the untreated fellow eye (*p* = 0.007%) [51]. The results of the larger Phase III DERBY (NCT03525613) and OAKS (NCT03525600) trials, are expected in the third quarter of 2021 [51].

### Integrin-targeting therapy

Risuteganib, (Luminate, ALG-1001, Allegro Ophthalmics, San Juan Capistrano, CA, USA), targets integrins αvβ3, αvβ5, and α5β1, all of which are receptors involved with angiogenesis. Risuteganib has completed four Phase 3 trials: one for dry AMD (NCT03626636); one for nAMD (NCT01749891), one for DMO (NCT02348918) trials; and one for the treatment of vitreomacular adhesion (NCT02153476). Results have been published for the DMO trial, DEL MAR, in which four doses of risuteganib were compared with bevacizumab [52, 53]. Risuteganib was administered at weeks 0, 4 and 8, with an as-needed further injection at week 20. Bevacizumab was administered every 4 weeks for the first three doses, then as-needed at weeks 12, 16, and 20. The primary endpoint of noninferiority compared to bevacizumab in terms of BCVA and improvement in central macular thickness was met at the 20-week time point. Allegro therapeutics had announced a Phase III trial of risuteganib in 2018, which are expected to start in late 2021/ early 2022.

### Rho-kinase inhibitors

Rho-kinase inhibition has become an attractive therapeutic target in ophthalmology. The upregulation of the Rho-kinase (ROCK) pathway has been shown, in diabetes, to promote angiogenesis and vasculopathy, thus inhibiting ROCK signalling could have beneficial effects in treating neovascular diseases of the retina, either alone, or in combination with other therapies. It also has a role in glaucoma therapy: netarsudil (Aerie Pharmaceuticals, Inc., Irvine, CA, USA) is a ROCK inhibitor marketed in Europe as Rhokiinsa, (and Rhopressa in North America) that is available as a 0.02% ophthalmic solution that is used to lower the IOP in ocular hypertension or open-angle glaucoma [54]. In this context, ROCK inhibition appears to both increase aqueous humour outflow through the trabecular meshwork and reduce venous pressure in the episcleral layer of the eye. Netarsudil is a prodrug and after topical administration, it is rapidly metabolised to its active metabolite, AR-13503 by esterases in the cornea [55]. AR-13503, formulated in a biodegradable sustained release implant that releases the drug over a 4–6 month period, is under early phase clinical investigation by Aerie as monotherapy for nAMD and DMO (NCT03835884). Another ROCK inhibitor, Fasudil (HA-1077, Asahi Kasei Pharma, Tokyo, Japan) has shown promise when administered in combination with bevacizumab in a pilot study performed in patients with DMO [56].

### Others

It has been observed that synonymous single nucleotide polymorphisms (SNPs) in the gene, *high-temperature requirement A1* (*HtrA1*) increase the inherited risk of nAMD [57]. *HtrA1* encodes a serine protease, and recently a potent anti-HtrA1 Fab inhibitor of HtrA1’s proteolytic activity, Fab15H6.v4.D221, has been developed, and a substrate (and biomarker) of HtrA1 activity, Dickkopf-related protein 3 (DKK3), has also been identified [58]. A small Phase 1 clinical study of the antibody in patients with GA secondary to AMD showed dose-dependent inhibition of DKK cleavage that lasted for over 8 weeks following a single intravitreal Fab15H6.v4.D221 injection [58].

### Implants

#### Port delivery system (PDS)

A different method of delivering effective drug doses over an extended period is to use a slow-releasing intraocular device such as the PDS device (Roche). The PDS is a permanent, refillable implant that is placed in the eye through a small incision in the sclera and pars plana. The PDS has a self-sealing septum in the centre of the implant flange which allows clinicians to refill the implant reservoir. The drug present in the PDS passively diffuses along a concentration gradient from the implant reservoir to the vitreous cavity, via a porous metal release control element. This approach is currently under investigation with not only ranibizumab [59], but also faricimab (NCT04567303)—and it is possible that the PDS refilling interval will be approximately 4 months or longer. The Phase III ARCHWAY study (NCT03677934) evaluated ranibizumab in patients with nAMD, administered monthly (at a concentration of 10 mg/mL) by intravitreal injection, or with the PDS implant filled with ranibizumab at a concentration of 100 mg/mL. It was reported that 98.4% of patients in the PDS group were able to go 6 months without needing rescue treatment and at weeks 36–40, achieved similar mean BCVA gains to patients receiving monthly ranibizumab eye injections (0.2 and 0.5 letter gain from baseline, respectively) [60]. Results from Phase II of the LADDER trial of the PDS with ranibizumab for wet AMD showed comparable outcomes over 22 months with monthly intravitreal injections of the drug [59, 61]. A new Phase IIIb study (NCT04657289; not yet recruiting) aims to compare 24- and 36-week refill regimens for the PDS with ranibizumab, thus further decreasing the treatment burden on patients and improving medication adherence.

#### Corticosteroid treatments

Corticosteroids are a powerful tool for controlling ocular inflammation and act by reducing the expression of a wide range of pro-inflammatory and pro-angiogenic cytokines (including VEGF), and have successfully been used in treating DMO and uveitis. Their use in DMO is typically second-line to anti-VEGF therapy, as intravitreal steroid application can be associated with increases in IOP in some patients and accelerated cataract development in most patients who still have their natural crystalline lens [62], and anti-VEGF use is still the default for the subset of patients who may need to avoid steroid therapy. Recently, an expert panel established a consensus on the management of DMO using dexamethasone implants, favouring their utility in a variety of patient situations (pseudophakic, poorly adherent, candidates for cataract surgery, high inflammatory component, history of cardiovascular events, and more) [63]. Regular intravitreal injections can present adherence challenges for patients, thus the introduction of intravitreal implants offers a longer-lasting and potentially a more convenient option. They also demonstrate superiority in vitrectomised eyes, in which VEGF inhibitors are more rapidly cleared [64]. Patients who fail VEGF inhibitor therapy have shown functional and anatomic improvement after switching to steroid therapy [65].

##### Dexamethasone implant

An extended-release intravitreally implanted formulation of dexamethasone (Ozurdex, Allergan/ Abbvie, Irvine, CA, USA) was approved by the European Medicines Agency for the treatment of DMO in pseudophakes or patients who are considered insufficiently responsive to, or unsuitable for non-corticosteroid therapy, macular oedema following either branch or central RVO, or inflammation of the posterior segment of the eye presenting as non-infectious uveitis [66–69] The retreatment interval varies by indication: in DMO, it is recommended that retreatment is performed after ‘~6 months’, whereas retreatment in patients with RVO or uveitis ‘should be considered when a patient experiences a response to treatment followed subsequently by a loss in visual acuity and in the physician’s opinion may benefit from retreatment without being exposed to significant risk.’ In practice, clinicians use clinical markers of activity (recurrence of oedema and/or vision loss) to determine whether retreatment with dexamethasone implant is necessary, and that can be every 3–4 months.

##### Fluocinolone implant

A second steroid-releasing intravitreal implant, in this case, one that contains fluocinolone acetonide (Iluvien, Alimera Sciences, Alpharetta, GA, USA) is also available, and is reported to release fluocinolone acetonide for a period of up to 36 months. Its approved indications in Europe are ‘the treatment of vision impairment associated with chronic DMO considered insufficiently responsive to available therapies’ and the ‘prevention of relapse in recurrent non-infectious uveitis affecting the posterior segment of the eye.’ [70] Retreatment intervals ‘may be administered after 12 months if the patient experiences decreased vision or an increase in retinal thickness secondary to recurrent or worsening DMO’, only ‘if the potential benefits outweigh the risks.’ [70] Its effects on uveitis [71–73] and DMO [74, 75] have been well characterised in late-phase clinical trials.

##### Suprachoroidal administration of triamcinolone acetonide

Suprachoroidal administration of triamcinolone acetonide is associated with the drug diffusing through the suprachoroidal space, which concentrates the drug mostly in the sclera, choroid and retina, and minimises the amount of drug that accumulates in the anterior chamber, lens and vitreous [76, 77] an approach that should help minimise the well-known off-target steroid adverse effects of cataract formation and IOP rises. Access to the suprachoroidal space has become easier with the development of microneedles that enable drug delivery by ophthalmologists in an outpatient setting, as opposed to sclerotomy or *ab interno* surgical approaches that were previously required [77].

A propriety, preservative-free formulation of triamcinolone acetonide for suprachoroidal administration, CLS-TA (XIPERE, Clearside Biomedical, Alpharetta, GA, USA), has undergone late-phase clinical evaluation for the treatment of DMO, and macular oedema secondary to non-infectious uveitis and RVO.

A Phase III trial of CLS-TA comparing CLS-TA in combination with aflibercept with aflibercept monotherapy was performed in 460 patients with RVO and macular oedema (SAPPHIRE, NCT02980874) was terminated after 8 weeks as no additional benefit of the combination therapy was seen at that point [78]. After this, Clearside Biomedical reported that they would discontinue the clinical development of CLS-TA for RVO and focus on its development as an uveitis monotherapy [78].

A 12 weekly dosing of CLS-TA (or sham treatment, randomised in a 3:2 ratio) was evaluated in 160 patients with macular oedema secondary to non-infectious uveitis for a 24-week period in the Phase III PEACHTREE trial (NCT02595398). Suprachoroidally administered CLS-TA was significantly better than sham treatment at achieving ≥15 letter BCVA gains from baseline (47% vs. 16%, *p* < 0.001) and mean reductions in central subfield thickness (CST) from baseline (153 µm vs. 18 µm, *p* < 0.001). Steroid-associated elevations in IOP rates and cataract development rates were 11.5 and 15.6%, and 7.3 and 6.3% [79].

### Steroid-sparing anti-inflammatories in uveitis

Many patients with non-infectious uveitis affecting the posterior segment will receive systemic corticosteroid therapy, particularly in the acute setting. This can be very effective, but typically multiple systemic side effects accumulate as the duration of therapy lengthens. Local steroid treatment as described above can become an option for some patients, but immunosuppressant drugs are also an option that can help reduce long-term corticosteroid dependence. These agents include antimetabolites, T-cell inhibitors, alkylating agents, and biologics targeting B- and T-cell activation, interferon therapy, and the use of drugs that interfere with the action of interleukin-6. Many of these therapies require frequent dosing and so are outside the scope of this review, but some require infrequent administration, for example, rituximab (MabThera, Rituxan, Roche) and alemtuzumab (Campath/Lemtrada, Sanofi, Paris, France).

Rituximab is a chimeric monoclonal antibody which blocks CD20, a cell surface marker found on B-lymphocytes [80]. Rituximab is commonly administered initially as two intravenous doses given 2 weeks apart, then approximately every 6 months, depending on the initial response to therapy and the recurrence of disease activity. Most clinical experience with rituximab is derived from its use in patients with rheumatoid arthritis, and these data suggest the risk of a serious infusion reaction is <1%, and infection rates have been estimated at around 2–3% [80]. However, the evidence base for its efficacy in uveitis is more limited with only a series of case reports [81, 82], and two Phase I/II trials for the treatment of orbital inflammation and refractory scleritis [83, 84]. Of the 10 patients enroled in the orbital inflammation trial, seven showed improvement with rituximab infusions [84], and of the 12 involved in the scleritis, eight responded to the therapy [83]. Rituximab has also been used off-label for the treatment of retinal vasculitis or myelitis in patients with Behcet’s disease [85–88].

Alemtuzumab is a monoclonal antibody that binds CD52 (a protein that is expressed on mature lymphocytes) and targets these lymphocytes for destruction. Its principal indications are for the treatment of B-cell chronic lymphocytic leukaemia, and as a third-line treatment for relapsing-remitting multiple sclerosis [89]. Alemtuzumab is parenterally administered as daily infusions: daily for 5 consecutive days for the first dose, then daily for 3 consecutive days for the second and subsequent doses. However, the interval between each course of infusions is 12 months [89]. The evidence base for alemtuzumab in uveitis is limited; with a case report of its intravenous administration in a 17-year-old patient with active relapsing multiple sclerosis and bilateral optic neuritis (and subsequently bilateral intermediate uveitis and secondary macular oedema). The intraocular inflammation, previously refractory to conventional immunosuppressants, responded to alemtuzumab, inducing remission [90]. A clinical trial that involved 334 people with relapsing-remitting multiple sclerosis were randomised to receive either subcutaneous interferon beta-1a (IFNB-1a), alemtuzumab 12 mg or alemtuzumab 24 mg [91]. Visual contrast sensitivity assessments were performed on all eyes at baseline, and every 3 months after treatment up to 36 months of follow-up. At 3 months, alemtuzumab-treated patients were significantly more likely (*p* = 0.013) to experience visual improvement of at least 0.3 log units, and at the end of the follow-up period, the contrast sensitivity gains (pooled alemtuzumab: 0.080 log unit gain vs. IFNB-1a 0.038 log unit gains) were still present (*p* = 0.0102).

However, these therapies are not licensed in the United Kingdom, nor are they funded for use in the National Health Service, and it is important to note that in 2018, alemtuzumab was subject to a FDA Safety Announcement, that noted ‘rare but serious’ instances of stroke and blood vessel wall tears in patients with multiple sclerosis, some of which were fatal, and most of which occur within 1 day of treatment initiation [92]. In 2019, the EMA’s Pharmacovigilance Risk Assessment Committee started a review of alemtuzumab following new reports of immune-mediated conditions and of problems with the heart and blood vessels with this medicine, again including fatal cases [93].

#### Gene therapy

Gene therapy holds considerable and transformative potential for the development of therapies that could significantly reduce treatment burden. For example, gene therapy can potentially be used to generate long-term therapeutic biological molecule production in the eye. The complexity of retinal disease pathogenesis can make the therapy more challenging to be administered, and for treatment to be successful, several factors need to be addressed including the definition of the therapeutic window, safe and efficient vectors, identification of a suitable target gene, and a reliable means of regulating transgene expression, as well as patient selection and outcome measurement.

RGX-314 is a one-time, subretinal gene therapy that uses an adenoviral vector to introduce a monoclonal antibody fragment into the eye. The antibody neutralises VEGF to reduce or eliminate abnormal blood vessel growth, has been shown to reduce anti-VEGF treatment burden, and appears to be well-tolerated as a subretinal injection [94]. The Phase II AAVIATE trial (currently recruiting) will randomise patients to receive either RGX-314 in the suprachoroidal space or control treatment (ranibizumab), thus evaluating the efficacy of a potential one- or two-dose treatment for nAMD [95]. ADVM-022 (Adverium Biotechnologies Inc., San Francisco, CA, USA) utilises a propriety vector capsid, AAV.7m8, which carries an aflibercept coding sequence under the control of a proprietary expression cassette, and preliminary results of the OPTIC trial (NCT03748784) of two doses of ADVM-022 in patients with nAMD suggest that stable aflibercept production is present at up to 30 months post-administration, with the 14/15 patients who received the higher ADVM-022 dose required no rescue anti-VEGF rescue therapy (six patients with 84 weeks follow-up; six patients with 16 weeks follow-up) [96].

GT005 is another potential AAV-2 based gene therapy, this time for dry AMD. It is delivered as a one-time treatment into the suprachoroidal space in patients with GA secondary to dry AMD. GT005 upregulates complement factor I (CFI), which counters inflammation caused by an overactive complement system. The HORIZON trial (currently recruiting) will randomise patients to receive either GT005 (medium or high dose) or no treatment, thus evaluating the efficacy of a potential single-dose treatment for dry AMD [97]. The open-label Phase I/II FOCUS study recently has reported a sustained increase in CFI with single suprachoroidal delivery of GT005 [98].

### Artificial intelligence

The combination of the plethora of treatment options under development, and the fact that many of them have novel (and even multiple) mechanisms of action can make choosing the optimal treatment option for patients challenging. There is much interest in the potential application of artificial intelligence (AI) in terms of automated diagnostic screening. Further to the benefits that this will bring, an additional key step in optimising treatment in these different conditions will be to integrate the prediction of the best personalised treatment plans by analysing response to agents used, evaluating dosing regimens in big datasets. This will enable us to achieve the best possible outcomes with the lowest patient and healthcare system burden [99].

### Glaucoma

Glaucoma is a common, currently irreversible cause of vision loss, characterised by optic nerve head excavation and loss of visual field. The mainstay of glaucoma therapy remains medical treatment in the form of eye drops, although adherence and persistence (i.e. continued correct compliance) have been found to be suboptimal, even when compared with other chronic medical conditions. As a result, longer-acting therapies for glaucoma are of significant interest (Table 3).

**Table 3:** Non-anti-VEGF long-acting retina and glaucoma pharmacotherapies.

	**Type**	**Mechanism**	**EMA-approved ophthalmic indication in adults**	**EMA-approved dosing regimen**
Dexamethasone (Ozurdex)	Glucocorticoid	Agonist of the glucocorticoid receptor	DMO, RVO, uveitis	Single-use biodegradable implant lasting 3–4 months
Fluocinolone acetonide (Iluvien)	Glucocorticoid	Agonist of the glucocorticoid receptor	DMO, uveitis	Single-use biodegradable implant lasting ≤36 months
				**Investigated regimens**
Triamcinolone acetonide (CLS-TA; Xipere)	Glucocorticoid	Agonist of the glucocorticoid receptor	N/A	12 weekly dosing for the treatment of noninfectious uveitis.
Risuteganib (Luminate)	Anti-integrin peptide	Targets integrins αvβ3, αvβ5, and α5β1	nAMD, DMO	nAMD: Initially q4w for three doses, then at week 20 as needed
GB-102	Sunitinib maleate polymer nanoparticle extended-release formulation	Tyrosine kinase inhibitor	N/A	nAMD: 6-monthly dosing
OTX-TKI	Axitinib intravitreal implant, suprachoroidally administered	Tyrosine kinase inhibitor	N/A	nAMD: 12-monthly dosing
PAN-90806	Topically administered tyrosine kinase inhibitor suspension that reaches the retina via the trans-scleral vascular route.	Tyrosine kinase inhibitor	N/A	Daily topical drops (which may extend anti-VEGF intravitreal injection intervals to ≥3 months).
GT005	Recombinant non‐replicating AAV vector encoding human complement factor I (CFI)	Addresses depletion in CFI levels causing complement dysfunction	N/A	nAMD: single-dose under evaluation
Bimatoprost Implant (Durysta)	Analogue of prostaglandin F_2α_	Ester prodrug; increases outflow of aqueous fluid from the eye; does not act on any known prostaglandin or prostamide receptor; believed to work via trabecular meshwork and uveoscleral pathways	Glaucoma	Single-use biodegradable implant lasting 3–4 months under evaluation^a^
Travoprost XR	Analogue of prostaglandin F_2α_	Ester prodrug; increases outflow of aqueous fluid from the eye; does not act on any known prostaglandin or prostamide receptor; believed to work via trabecular meshwork and uveoscleral pathways	Glaucoma	Single-use biodegradable implant lasting 3–4 months under evaluation; single-use punctal plug lasting 90 days under evaluation

*DMO* diabetic macular oedema, *BRVO* branch retina vein occlusion, *CNV* choroidal neovascularisation, *CRVO* central retinal vein occlusion, *Fab* fragment, antigen binding, *GA* geographic atrophy, *Ig* immunoglobulin, *m* month, *nAMD* neovascular age-related macular degeneration, *PRN* pro re nata (as needed), *q* *quaque* (every), *w* weeks, *AAV* adeno-associated virus.

^a^FDA-approved regimen; not yet approved by the European Medicines Agency.

One approach has been the development of biodegradable implants, such as Bimatoprost SR (Durysta; Allergan, Dublin, Ireland) [100–102]. This utilises the Novadur (Allergan, Dublin. Ireland) biodegradable drug-delivery system which has been used as a delivery system for dexamethasone (Ozurdex; Allergan, Dublin, Ireland) since 2009 [103]. The rod-shaped implant is administered with a single-use 28-gauge applicator and provides a non-pulsatile, continuous release of bimatoprost for between 3 and 4 months [102]. Initial IOP-lowering data came from a 24-month Phase I/II clinical trial, APOLLO, which found comparative IOP-lowering at a range of doses when compared to once-daily topical bimatoprost 0.03% [100, 101]. Later Phase III trials, ARTEMIS-1 and ARTEMIS-2, assessed the efficacy and safety of 10- and 15 µg implants in adults with either POAG or ocular hypertension (OHT) with open inferior angles and baseline IOP of 22–32 mmHg after a washout period [102]. Both tested doses were non-inferior to twice-daily topical timolol maleate 0.5% at 12 weeks. In ARTEMIS-1, at 52 weeks follow-up (after administration at three fixed 16-week intervals), 84.3% (167/198) of subjects receiving the 10 µg implant had not required further IOP-lowering treatment [102]. The FDA-approved Durysta in March 2020 for use in patients with POAG or OHT, currently as a one-off treatment given the potential risk for corneal endothelial cell loss.

Travoprost has also been utilised in drug-eluting intracameral implants. Travoprost XR (Aerie Pharmaceuticals, USA) is a biodegradable implant that uses novel sterile nanoparticle replication engineering technology to provide continuous release of travoprost. A 12-month study of 15 patients with POAG demonstrated noninferiority to twice-daily timolol maleate 0.5% at 11-month follow-up, with a mean IOP reduction of 6.7 ± 3.7 mmHg [104]. The OTX-TIC implant (Ocular Therapeutix, USA) contains micronised travoprost released over 4–6 months. Initial Phase I trial data with 6-month follow-up showed a greater IOP-lowering effect compared to the fellow eye treated with once-daily topical travoprost [105]. An ongoing study of additional cohorts is underway to provide longer-term data. The iDose Travoprost implant (Glaukos, San Clemente, CA, USA) is a titanium intracameral delivery system that is anchored to the trabecular meshwork. The implant is encased in a membrane that provides continuous drug elution and can be replaced for ongoing treatment. A Phase II study found a similar IOP-lowering effect for two different elution rates compared with timolol maleate 0.5% at 12-week follow-up (NCT02754596). A Phase III trial has a primary completion date of June 2021.

A travoprost eluting punctal plug, OTX-TP (Ocular Therapeutix, USA) is a hydrogel rod that swells to fit the canalicular space and continuously releases travoprost over a 90-day period. A placebo-controlled multicentre Phase III trial found OTX-TP treated eyes had statistically significant IOP reduction in eight of nine time points over 20-weeks of follow-up, with only transient and minor adverse effects, most commonly dacyrocanaliculitis [106]. However, the system is reportedly no longer under development [107]. Other external routes recently considered have included topical forniceal inserts [108], subconjunctival injections [109], and micelle-laden contact lenses to achieve sustained drug release [110].

Longer-term IOP-lowering can also be achieved by laser treatment, most commonly as selective laser trabeculoplasty (SLT). First approved by the FDA in 2001, today SLT is widely used, more recently also as a first-line treatment option following the publication of the LiGHT study in 2019 [111]. As SLT uptake becomes more prevalent, more focus has been given to patient selection and duration of effect. A multicentre real-world analysis of 831 SLT-treated eyes found treatment success in 70%, 45% and 27% of treated eyes at 6-, 12- and 24-month follow-up respectively [112]. Pre-treatment IOP was found to be the most significant predictive factor for success, in line with findings in previous studies [112–114]. The SALT trial found post-procedure topical ketorolac 0.5% or prednisolone 1% may improve the IOP-lowering effect at 12 weeks follow-up, although longer-term effects require further investigation [115].

Finally, over the past decade, a series of novel surgical approaches to glaucoma have aimed to provide long-acting alternatives to topical medications for glaucoma, called minimally invasive glaucoma surgeries (MIGS). MIGS were intended to bridge the gap between medical or laser therapy and more invasive filtering surgery in mild-to-moderate glaucoma. They are meant to have a favourable safety profile ensuring prompt postoperative recovery and a reliable (but more modest) IOP reduction than that of traditional filtering surgery. Given that most of these devices have been introduced within the last 5 years, there is a lack of long-term data regarding their effectiveness, with most published evidence being limited to nonrandomised studies and uncontrolled retrospective comparisons, with few high-quality randomised controlled trials.

Longer-acting glaucoma treatments have the potential to address well-known adherence and persistence issues, reduce burden and cost on healthcare providers and remove the need for daily dosing for patients with glaucoma. More widespread usage of SLT appears likely going forward, given its low adverse effect profile. Intracameral drug-eluting systems hold promise, although concerns over damage to the corneal endothelium persist.

## Conclusion

Many new long-acting treatment options are currently in the pipeline, and although not all molecules will make it to the market, it is likely that in the next 5 years a number will do so, and will offer the promise of 12 weekly or longer treatment intervals, translating to around four treatments per year for patients. This reduced treatment burden is clearly going to benefit patients, healthcare providers and healthcare systems—and ultimately society too. Gene therapy holds a real potential to provide long-term benefits for patients, although there are several technical and cost hurdles that need to be overcome before this approach becomes widely available. We also need to address the fact that fewer clinic visits for treatment can also mean fewer opportunities for monitoring disease progress—particularly in fellow eyes—and also for recognising any ocular side effects that might be associated with these new agents. This is an issue that may be dealt with by creating imaging hubs or self-monitoring devices to provide regular assessments. It is anticipated that this would be supported by AI implementation in reading centres to assess the risk of disease activity or progression and treatment would be tailored accordingly. The advent of longer-acting therapies brings new treatment options to the horizon, many of which employ novel mechanisms of action. Finally, in addition to improving patients’ regimen compliance, the personalisation of these long-acting treatments to patients’ own specific disease biomarkers should allow better vision outcomes to be achieved, all with a lower treatment burden for all.
